# Soft Tissue Surgical Procedures for Optimizing Anterior Implant Esthetics

**DOI:** 10.1155/2015/740764

**Published:** 2015-06-01

**Authors:** Andreas L. Ioannou, Georgios A. Kotsakis, Michelle G. McHale, Donald E. Lareau, James E. Hinrichs, Georgios E. Romanos

**Affiliations:** ^1^Advanced Education Program in Periodontology, University of Minnesota, 515 Delaware Street SE, Minneapolis, MN 55455, USA; ^2^Private Practice, Edina, MN 55435, USA; ^3^Department of Periodontology, School of Dental Medicine, Stony Brook, NY 11794, USA

## Abstract

Implant dentistry has been established as a predictable treatment with excellent clinical success to replace missing or nonrestorable teeth. A successful esthetic implant reconstruction is predicated on two fundamental components: the reproduction of the natural tooth characteristics on the implant crown and the establishment of soft tissue housing that will simulate a healthy periodontium. In order for an implant to optimally rehabilitate esthetics, the peri-implant soft tissues must be preserved and/or augmented by means of periodontal surgical procedures. Clinicians who practice implant dentistry should strive to achieve an esthetically successful outcome beyond just osseointegration. Knowledge of a variety of available techniques and proper treatment planning enables the clinician to meet the ever-increasing esthetic demands as requested by patients. The purpose of this paper is to enhance the implant surgeon's rationale and techniques beyond that of simply placing a functional restoration in an edentulous site to a level whereby an implant-supported restoration is placed in reconstructed soft tissue, so the site is indiscernible from a natural tooth.

## 1. Introduction

Implant dentistry has been definitively established as a predictable treatment modality for replacing missing or nonrestorable teeth which yields excellent clinical success rates. During the last decade, the focus of implant research has shifted from the functional stability of the implant to its esthetic integration in the smile. The esthetics of implant restorations is dictated by two fundamental components: the reproduction of the natural tooth characteristics on the implant crown and the establishment of a soft tissue housing that will intimately embrace the crown. Therefore, the success of implant rehabilitation in the esthetic zone relies heavily on the preservation or the augmentation of peri-implant soft tissue by means of periodontal surgical procedures.

The aim of this paper is to enhance the implant surgeon's armamentarium with rationale and techniques that extend beyond the placement of a functional restoration in an edentulous site to the restoration of soft tissue harmony so that the implant-supported restoration is indiscernible from a natural tooth. This is especially important in areas of esthetic concern but not negligible in posterior sites where the added benefits of enhanced tissue contours cannot be overlooked.

## 2. Indications

It may not be an overstatement that every surgical implant procedure in the esthetic region constitutes an indication for soft tissue grafting. The inevitable alteration of the alveolar ridge dimensions that follows a tooth extraction often results in the placement of the implant in a site that has undergone a reduction in soft and hard tissue volume in comparison to its neighboring dentate sites [1–3]. This discrepancy is even more pronounced in single-implant sites where a concavity forms between the edentulous site and the root prominences of the neighboring dentition. Subepithelial connective tissue grafts (SCTG) or free gingival grafts (FGG) can be employed in these cases to reconstruct the buccal dimensions of the site improving the tissue thickness. In addition, they create the illusion of root prominence and increase the width of the crestal peri-implant mucosa in order to provide an emergence profile for the restoration and enable the constructed site to closely resemble a natural tooth.

The long-term stability of pink esthetics around dental implant prostheses has been strongly correlated with adequate peri-implant soft tissue thickness, that is, a thick peri-implant biotype [4, 5]. When a thin biotype is diagnosed, a SCTG or a FGG can be used to prevent potential long-term recession of the facial mucosal margin or permeation of a gray color from the implant [6–8].

**Figure 1:**
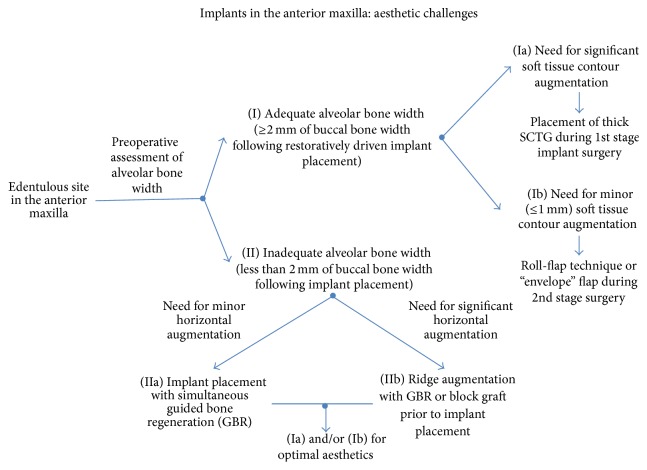
Implants in the anterior maxilla: a clinical decision-tree for overcoming aesthetic challenges.

Factors that should be considered when evaluating the need for soft tissue grafting include the level of clinical attachment on adjacent teeth to support papillary height, the thickness of the coronal soft tissue margin to ensure a proper emergence profile, the thickness of labial soft tissue to simulate root eminence and prevent transillumination of underlying metallic structure, and the position of the mucogingival junction and amount of keratinized tissue so as to blend harmoniously with that of the adjacent teeth [9, 10] (Figure 1).

## 3. Contraindications and Limitations

General and specific limitations apply to the use of a soft tissue augmentation technique around dental implants. Certain medical conditions are considered general contraindications to surgical intervention. Collagen disorders, such as erosive lichen planus and pemphigoid, may pose a risk to the viability of autogenous connective tissue grafts placed on a recipient bed that exhibits a pathologic healing response. There is no published evidence to either support or discourage the use of soft tissue grafting techniques in such cases.

Smoking is another relative contraindication. It is well established that a key determinant of soft tissue augmentation success is revascularization of the graft. Nicotine contained in cigarettes causes vasoconstriction to the surgical site, often resulting in necrosis of the graft [11]. This nicotine-associated vasoconstriction, in combination with lack of adherence of the fibroblasts [12] and alteration in immune response [13, 14], diminishes the likelihood for a successful outcome. Preoperative assessment should attempt to identify such at-risk patients whereby the clinician must inform the patients of the potential adverse effects associated with smoking. Local factors that may also limit patient selection include lack of adequate tissue thickness at the palatal donor site or restricted surgical access to intraoral donor sites such as the posterior of the hard palate or maxillary tuberosity.

## 4. Treatment Planning and Timing for Soft Tissue Grafting Procedures

A thorough 3-dimensional preoperative evaluation of the edentulous site is critical to properly planning an implant case that will result in an esthetic outcome. Two diagnostic variables that should be taken into account preoperatively are bone and soft tissue volumes [15]. Long-term stability of esthetics for an implant requires the implant to be surrounded by ~1.8–2.0 mm of vital bone [16]. Lack of adequate bone necessitates hard tissue grafting. Sites should also be evaluated for soft tissue profile. A discrepancy of soft tissue contours with adjacent teeth can be addressed with augmentation.

Soft tissue augmentation can be performed simultaneously with implant placement and/or during the second stage surgery, as will be described in the following technique section. There is no evidence in the literature to support any advantage of simultaneous soft tissue augmentation over augmentation during second stage surgery. Both treatment modalities have been shown to lead to better esthetics and increased soft tissue thickness [17]. Even though both techniques yield favorable esthetics, the earlier the intervention is performed, the more opportunities the clinician has to better control the final outcome. For instance, in a case where the residual ridge has undergone significant atrophy, the simultaneous soft tissue augmentation in conjunction with first stage surgery will allow sufficient healing time to properly assess the site during second stage surgery. Consequently, additional soft tissue augmentation can be performed simultaneously when uncovering the implant(s) in order to achieve a more ideal outcome.

Soft tissue grafting can also be utilized as a “rescue procedure” to manage esthetic complications associated with implants. Labial inclination of implants, buccal placement, or use of wide body contributes to a thin tissue biotype or thin buccal bone that may lead to recessions [18], permeation of gray from the implant structure through the tissue, and exposure of the titanium implant neck, all of which contribute to an inharmonious emergence profile of the implant-supported restoration and an ersatz appearance of the patient's smile [19, 20]. Additionally, soft tissue grafting following implant placement can be used to correct complications associated with soft tissue color mismatch to a level below clinical perception [21].

## 5. Free Gingival Graft

The use of autogenous FGG in mucogingival surgeries predates that of any other type of graft. FGGs are considered a reliable and efficacious approach for augmenting peri-implant soft tissue defects and are most often utilized to increase the amount of keratinized tissue around an implant. FGGs are the gold standard in cases when an increase in keratinized tissue is desired.

**Figure 2:**
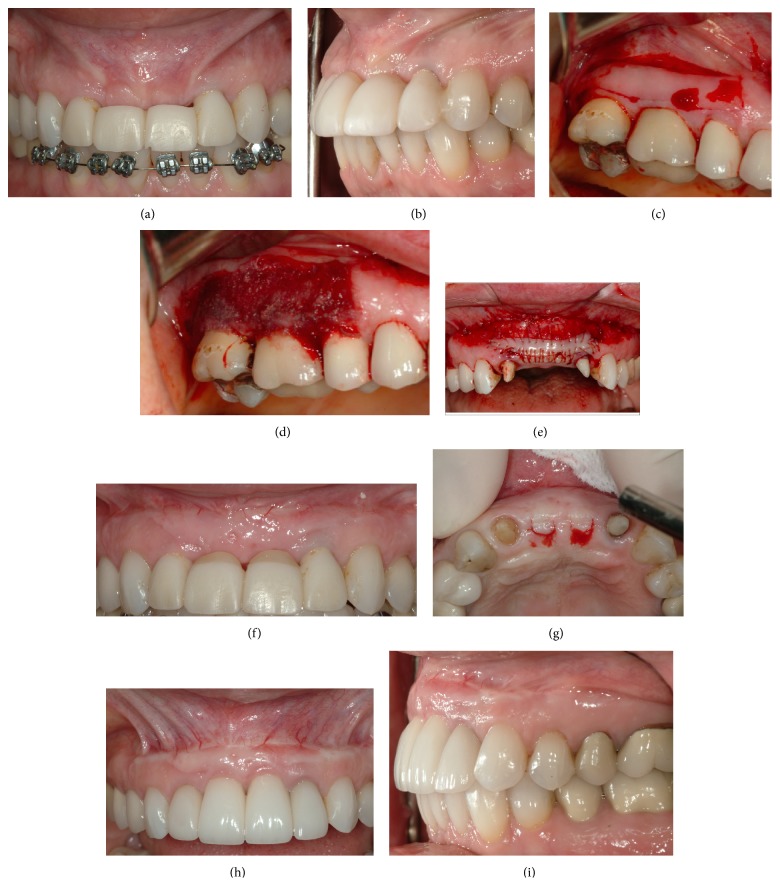
(a) Patient had previous bone grafting and numbers 8 and 9 implant placement. Note minimal keratinized attached gingiva over grafted area of numbers 8 and 9 due to coronal advancement of the flap. (b) Note the deficient soft tissue profile following placement of a provisional prosthesis with appropriate tooth emergence. (c) Donor site and graft procurement. (d) Collagen tape and cyanoacrylate to reduce discomfort over donor site. (e) Graft secured and well adapted to recipient bed with multiple sutures. (f) Recipient site following healing. Note the increase in height and thickness of the keratinized attached gingiva. (g) Numbers 8 and 9 implant sites prepared for second stage surgery. (h) Recipient site after numbers 8 and 9 implant restorations, showing stable keratinized attached gingiva. (i) Lateral view of recipient site. Note the thick buccal keratinized attached gingiva, establishing an esthetic emergence profile for the implant restorations.

The most common donor site of a FGG is the highly keratinized hard palate. That being said, the color and shade of the augmented recipient site do not often blend naturally with the adjacent soft tissues. This produces a nonesthetic result, contradicting the initial purpose of the procedure. Even so, a FGG to increase the keratinized tissue is recommended for “rescue” procedures to cover exposed implant threads. In addition, a FGG can be used for patients with low smile lines, when extensive soft tissue augmentation is needed, or where the color of a FGG will not compromise the esthetic appearance of the implant site (Figure 2).

## 6. Subepithelial Connective Tissue Graft

**Figure 3:**
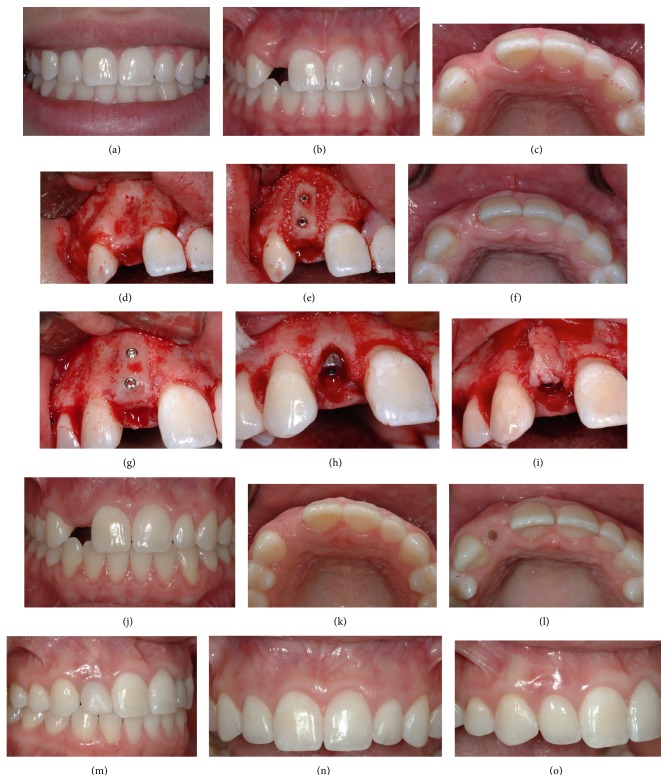
((a), (b), and (c)) Patient presented for implant rehabilitation of number 7 lateral incisor. Not the high interdental smile line that poses an esthetic challenge. Following ridge resorption, a concavity consistent with a Seibert Class I defect is seen in the edentulous site. ((d), (e), and (f)) A block autograft was screwed in place to achieve horizontal ridge augmentation prior to implant placement. Particulated allograft was utilized to graft the area between the block and the recipient bed. Note the significant enhancement of the tissue profile postsurgically. ((g), (h), and (i)) At four months after grafting the site was reentered and an implant was placed in the ideal 3-dimensional position. A SCTG was utilized to replicate the root eminence and provide a natural emergence profile. ((j), (k), (l), and (m)) Postoperative healing view shows excellent tissue contours at the site. A customized healing abutment was selected to mold the tissues after 2nd stage surgery. Note the excellent positioning of the mucosal zenith at the time of provisionalization. ((n), (o)) Intraoral view of the final restorations in place. Crown lengthening was performed on the adjacent teeth to address the patient's overall esthetic demands. Note the excellent replication of gingival characteristics on the peri-implant mucosa and the natural appearance of the restoration as it emerges from the augmented hard on soft tissues at the site.

SCTG procedures have been used successfully throughout the years for the management of recession and soft tissue defects around natural teeth and for augmenting alveolar ridge contours [22, 23]. Some may argue that the traditional approaches for connective tissue grafting do not fare well when one attempts to graft and achieve cover of a nonvital implant surface since the soft tissues around the implant do not respond in the same manner as a vital tooth. Nonetheless, many of these procedures can be translated directly to peri-implant soft tissue modification and esthetic optimization. When indicated and properly utilized, these surgical procedures can provide stable and significant gains in soft tissue volume and contour that can contribute to the successful esthetic management of implant sites (Figure 3).

## 7. Technique for Soft Tissue Grafting during 1st Stage Implant Surgery

*Step  1: Treatment Planning*. As in all surgical procedures, treatment planning is the cornerstone of success. Preoperative identification of potential soft and/or hard tissue deficiencies allows for the construction of an implant restoration that will closely mimic that of the natural dentogingival complex and blend with the existing dentition in a pleasing and esthetic fashion. A decision should be made preoperatively whether soft tissue augmentation alone will be adequate to develop the desired treatment outcome or if bone augmentation is also needed to achieve ideal implant position and soft tissue esthetics.

*Step  2: Graft Harvesting*. The three most common intraoral donor sites for harvesting connective tissue grafts are the tuberosity [24], the single incision-deep palatal [25], and the free gingival graft method-superficial palatal [26]. Donor tissue for FGGs is routinely harvested from the hard palate since this area provides an ample surface area of keratinized tissue. Nonetheless, relatively any intraoral site with adequate tissue thickness that displays keratinization, such as the keratinized epithelium apical to the gingival crest of the maxillary molars, may be utilized to procure a FGG. The amount and quality of soft tissue available for harvesting depend on donor site, that is, tuberosity versus palate. The tuberosity generally provides enough tissue to cover a single or two implant site(s), while adequate tissue can be obtained from the palate to cover an area two or three times wider than that of the tuberosity, depending on the incision design. The quality of the tissue harvested from the tuberosity is superior to that obtained from the palate since the tuberosity offers a graft composed of dense connective tissue, whereas the portion of the palatal connective tissue donor usually consists of adipose tissue. Tissue obtained from the tuberosity usually permits the harvesting of a significantly thicker graft than that obtained from the palate [27]. This broad piece of tuberosity can be longitudinally sectioned to increase the amount of donor tissue.

### 7.1. Harvesting from the Tuberosity

On the distal aspect of the tuberosity a single, crestal beveled incision is made from the mucogingival junction to the distofacial line angle of the most distal tooth. The incision is located on the buccal aspect of the ridge crest rather than midcrestal and connected to the distal surface of the most posterior tooth via a sulcular incision. Use of an Orban knife enhances the access to performing the sulcular incision. At this point, the palatal flap is raised until the distopalatal surface of the most distal tooth is exposed. Then, a new blade (15c) is used to meticulously dissect the connective tissue from the flap and the underlying periosteum. Tissue forceps and the suction tip should be delicately employed during procurement of the graft in order to minimize excessive trauma to the donor tissue and prevent inadvertent loss of the graft through the suction tip. Once the graft has been obtained, it is stored in saline to prevent dehydration while the recipient bed is prepared. The donor site flap is sutured closed at this time, preferably using 4-0 chromic gut and a continuous interlocking suturing technique.

### 7.2. Harvesting from Deep Palatal Tissue

If a deep palatal donor site is selected for harvesting the connective tissue graft, the donor site should be sounded to bone. This is performed to verify that the incision will not involve a periodontal pocket or bony dehiscence of a palatal root in order to avoid postoperative recession. A single, full-thickness horizontal incision is made at a right-angle to the alveolar bone of the palatal keratinized tissue approximately 3 mm from the free gingival margin of the maxillary teeth. This first incision extends from the mesial aspect of the palatal root of the maxillary first molar as far anteriorly as needed for the appropriate amount of donor tissue required. A second incision is made parallel to the underlying bone so that a thin split-thickness flap is created to separate the underlying connective tissue from the superficial flap. When the desired volume of SCTG has been identified, the blade is directed towards the bone at the edges of the graft so that the SCTG is free except for its periosteal attachment. A Woodson elevator is slid under the partial-thickness flap to separate the graft from the underlying bone. The procured graft is kept in saline-soaked gauzes until used. The palatal flap can be closed with either single interrupted sutures, sling sutures around the maxillary teeth, or a combination of the above. It is important that the clinician be familiar with the anatomy of the palate in order to minimize the risk of hemorrhage associated with traumatizing the major palatine artery during harvesting of the graft. The arterial vascular trunk is typically located ~12–17 mm from the CEJ of the posterior teeth in patients with an average or high palatal vault while the artery is usually within 7 mm of the CEJ in patients with a shallow palatal vault [28].

### 7.3. Harvesting from the Superficial Palatal Tissue

This technique is used for the harvesting of both the FGG and the SCTG. This technique utilizes a very similar method to that of a FGG to harvest the SCTG, with the only difference being that the epithelium is removed after harvesting. The rationale for using this technique is that sounding reveals a limited amount of connective tissue beneath the palatal mucosa. In contrast to the tuberosity area where connective tissue occupies the whole tissue volume underneath the epithelium, here a limited amount of connective tissue exists between the epithelium (superficial) and adipose tissue (deep). Consequently, use of the deep palatal harvest technique in patients with thin palatal mucosa as described before would not procure an adequate thickness/volume of graft after removal of the adipose tissue.

The superficial palatal harvest technique places a horizontal anterior/posterior incision 3 mm away from the maxillary teeth, as described in the deep palatal harvest technique, as a partial-thickness incision of only 1.5–2 mm in thickness and leaves the periosteum intact. A second anterior/posterior horizontal partial-thickness incision is traced parallel to the first incision at a position closer to the midline. The distance between these two incisions is based upon the estimated amount of tissue graft required for grafting. The two horizontal incisions are connected via anterior and posterior vertical partial-thickness incisions on the mesial and distal aspect of the graft. Either a sharpened gingivectomy knife (Kirkland knife) or a blade (15c) is utilized to separate the graft from the underlying tissue for an ideal thickness of 1.5 mm to 2 mm. Then the graft is placed on a moist, sterile surface whereby the superficial epithelium is removed by sharp dissection. Adipose tissue is removed from the periosteal side of the graft with the aid of a fresh blade or LaGrange scissors until the harvested graft consists of only connective tissue or/and epithelium. The tissue graft is used as a template to trim a collagen biomaterial in the proper dimensions to cover the donor site wound. After adequate hemostasis has been achieved at the denuded donor site by application of gauze with digital pressure for 5–10 minutes, the collagen biomaterial is placed over the wound and secured by the application of cyanoacrylate via pipette. Periodontal dressing may be utilized depending on the surgeon's preference to improve patient comfort.

*Step  3: Preparation of the Recipient Site*. The flap is designed to retain a band of keratinized mucosa on the buccal aspect of the flap whenever possible. Consequently, it may be advisable to place the initial incision slightly palatal rather than midcrestal. The crestal incision is extended as sulcular incisions onto the adjacent teeth or as papillae sparing vertical releasing incisions passing to the level of the mucogingival junction. The length of each incision depends on the individualized treatment plan. A full-thickness flap is raised to allow access for surgical placement of the implant(s). The successful incorporation of a tissue graft does not depend on the thickness of the incision since the combination of a tissue graft with either a full- or partial-thickness flap yields similar clinical results [29]. The recipient bed should be kept well-hydrated with frequent irrigation throughout the procedure.

In order to create a partial-thickness flap, the dissection should occur beyond the mucogingival junction, leaving a layer of approximately 2 to 3 mm of connective tissue and periosteum intact.

*Step  4: Adaptation of the Soft Tissue Graft*. Following placement of the implant(s), the procured graft is adapted to the area. The dimensions of the graft should be adequate to provide soft tissue bulk at the level of the neck of the implant to ensure an esthetic emergence profile for the restoration as well as simulate a root prominence for the missing tooth. The tissue graft should be trimmed to resemble a semicircular cone so that the apical aspect does not span to the proximal surfaces of adjacent teeth. Such excessive soft tissue will create a bulky visual effect rather than that resembling the natural gingival contours of adjacent teeth. There is no significant clinical difference in regard to the orientation of the SCTG during its placement into the recipient site. Based on studies on root coverage procedures, when the periosteal side of the graft opposes the flap rather than the recipient bed, the success of the outcome will not be compromised [30].

*Step  5: Suturing at the Recipient Bed*. After trimming the graft to the appropriate dimensions, the graft is secured in the recipient bed utilizing a palatal-locking suture technique. The suture needle initially penetrates the palatal keratinized tissue in a palatobuccal direction. The needle then passes through the mesial aspect of the graft employing a faciopalatal direction. The sequence is repeated for the distal portion of the graft, and as the needle exits the palatal flap a second time, a knot is placed on the palatal side. The apex of the graft is stabilized in the connective tissue at the base of the flap so that the graft is stretched and well adapted onto the recipient bed. It is emphasized that the graft should be uniformly adapted and well secured on the recipient bed to prevent disruption of plasmatic circulation and healing. The final adaptation should be verified with the aid of a periodontal probe. Pressure is applied with moist gauze for 5 minutes. The flap is closed with single interrupted sutures using a 4-0 or 5-0 suturing material. If passive closure cannot be achieved, then horizontal vestibular releasing incisions should be placed in the base of the labial flap with a fresh 15C blade until tension-free flap adaptation and closure can be accomplished.

## 8. Technique for Soft Tissue Grafting during 2nd Stage Implant Surgery

A broad variety of techniques have been proposed to augment the soft tissue profile of implants at second stage surgery. Ideally, second stage surgery should be a minimally invasive procedure whereby minor revisions in soft tissue architecture can be accomplished to create a natural emergence profile for the healing abutment and/or final restoration [31]. A rolled pedicle flap can be used to augment the connective tissue that covers the coronal portion of a submerged implant. Tissue sounding is utilized to locate the palatal shoulder of the cover screw followed by an arcing crestal incision around the palatal aspect of the cover screw. Papillae sparing mesial and distal vertical releasing incisions are placed, leaving the labial pedicle flap intact. A blade (15c) is used to deepithelize the superficial layer of the labial pedicle flap. The labial pedicle is elevated as a full-thickness mucoperiosteal flap and a Woodson elevator is used to create a small tunnel beneath the base of the labial pedicle. A horizontal mattress suture with absorbable suturing material (5-0 chromic gut or vicryl) is initially passed from the base of the tunnel horizontally through the coronal margin of the deepithelized pedicle flap and back through the base of the tunnel in order to invert the deepithelized pedicle beneath the labial marginal gingiva. A knot is tied to secure the rolled pedicle flap beneath the labial pouch and can be verified by slight blanching of the area. The patient is instructed to avoid mechanical trauma to the area for the next couple of weeks and to use only a chlorhexidine rinse while the deepithelized pedicle flap heals. As in all implant cases, the construction of a well-contoured restoration is critical to the maintenance of a desirable soft tissue profile and an acceptable esthetic outcome.

Other minimally invasive techniques for contour augmentation are also available. One such example is the use of a buccal “envelope” technique for sliding a connective tissue graft on the labial aspect of the implant, as was originally described by Raetzke for use around teeth with mucogingival defects [32]. In this technique, sharp dissection is employed to produce a partial-thickness “envelope” flap that extends beyond the mucogingival junction on the facial of the implant [33]. Subsequently, a SCTG is procured and slid in the buccal envelope at the implant site. Lastly, sling sutures are utilized to secure the graft and coronally advance the flap [33]. Eghbali et al. have shown that a mean increase of 0.8 mm of mucosal thickness can be achieved with the use of this technique, whose increase is stable for at least 9 months after surgery. Therefore this procedure could be also considered in cases where minor buccal contour enhancement is indicated [33].

## 9. Conclusions

Implant dentistry has been established as a predictable treatment modality with high clinical success rates. Esthetic considerations for implant restorations and the role of surgical procedures in the creation and maintenance of peri-implant soft tissue have been gaining interest over the years. Clinicians who practice implant dentistry should attain more than just implant osseointegration to achieve an esthetic, successful outcome. Knowledge of the variety of techniques available and proper planning enable clinicians to meet patients' increasing esthetic demands. However, the need for soft tissue augmentation procedures around dental implants in the anterior esthetic zone remains a controversial topic and lacks support from the literature. Long-term clinical trials are needed for better assessment of these surgical procedures.
